# Towards a clinically practical computational platform for systematically adapting radiation therapy for glioma patients

**DOI:** 10.1088/1361-6560/ae9687

**Published:** 2026-08-24

**Authors:** Hugo J M Miniere, David A Hormuth, Ernesto A B F Lima, T J Whitaker, Maguy Farhat, Bikash Panthi, Holly Langshaw, Mihir D Shanker, Wasif Talpur, Sara Thrower, Jodi Goldman, Caroline Chung, Thomas E Yankeelov

**Affiliations:** 1 Biomedical Engineering, The University of Texas at Austin, Austin, TX 78712, United States of America; 2 Diagnostic Medicine, The University of Texas at Austin, Austin, TX 78712, United States of America; 3 UT Austin Cancer Research Center, The University of Texas at Austin, Austin, TX 78712, United States of America; 4 Oden Institute for Computational Engineering and Sciences, The University of Texas at Austin, Austin, TX 78712, United States of America; 5 Texas Advanced Computing Center, Austin, TX 78758, United States of America; 6 Radiation Oncology, The University of Texas MD Anderson Cancer Center, Houston, TX 77030, United States of America; 7 Radiation Physics, The University of Texas MD Anderson Cancer Center, Houston, TX 77030, United States of America; 8 Imaging Physics, The University of Texas MD Anderson Cancer Center, Houston, TX 77030, United States of America; 9 Faculty of Medicine, The University of Queensland, Australia

**Keywords:** glioma, radiotherapy, digital twin, mathematical modeling

## Abstract

*Objective.* High-grade gliomas (HGG) are aggressive brain tumors with poor prognoses despite an intense standard-of-care (SOC) chemoradiation protocol. Radiotherapy (RT) guidelines use static dose maps, which may not fully account for intratumoral heterogeneities and areas of tumor progression over the course of treatment. We address this limitation by guiding radiation delivery using imaging data to identify proliferative areas which are then targeted by a dose boost, and use mathematical modeling to simulate treatment response between the adjusted and the SOC treatment plan. *Approach.* Quantitative magnetic resonance imaging data for 15 HGG patients were collected at baseline and the third week of treatment to identify regions of high proliferation to guide the adapted radiation dose plan. The resulting dose plan is then adjusted by a board-certified medical physicist to ensure deliverability using clinically available linear accelerators. The plans are then used to virtually ‘treat’ each patient via our biology-based mathematical model, individually calibrated to each patient’s imaging data acquired prior to dose adaptation. *Main results.* Our adaptation pipeline predicted a median (range) decrease in tumor burden of 31% (12% and 57%) one month post-therapy across the patient cohort compared to the SOC predicted tumor burden (*p* < 0.05). After adjusting the dose plan for clinical deliverability, it still predicted a decrease in tumor burden of 30% (7% and 62%) which is also significantly different than the SOC protocol (*p* < 0.05). *Significance.* Our clinical-computational platform, integrating mathematical modeling, dose painting, and adaptive RT, can identify alternative radiation dose plans hypothesized to yield statistically greater tumor control than the SOC prescriptions.

## 1. Introduction

High-grade glioma (HGG) is the most common and the most aggressive primary brain tumor, accounting for approximately 14% of all brain malignancies (Ostrom *et al* 2021). The current standard-of-care (SOC) treatment follows the Stupp protocol, which prescribes maximal surgical resection of the tumor, followed by daily Temozolomide (TMZ) and radiotherapy (RT) given as 30 fractions of 2 Gy day^−1^, 5 d week^−1^ for 6 weeks), and finally adjuvant TMZ for 6 cycles of 28 d (Stupp *et al* 2005). Despite this aggressive treatment regimen, the prognosis of HGG is poor and has remained so for decades, with a median survival of approximately 15 months and a 5 year survival rate of 5% for its most aggressive form known as glioblastoma multiforme (Stupp *et al* 2017). Currently, RT is planned using a combination of x-ray computed tomography (CT) and magnetic resonance imaging (MRI) to locate the tumor and the surrounding critical structures. Medical physicists then use treatment planning software to design a personalized plan that delivers the prescribed radiation dose to the tumor while minimizing exposure to healthy tissues. The resulting dose map is then delivered over 6 weeks, without consideration for potential changes in local tumor biology or radiation sensitivity. Unfortunately, maintaining a static dose map throughout the entire course of RT fundamentally limits the treatment’s efficacy. Thus, there is a crucial need to better integrate changes in the tumor’s biology for treatment planning to extend patient survival.

There has been increasing interest in developing biologically-based assays for targeting radiation to expected areas of invasion or treatment resistance (Badiyan *et al* 2014, Nguyen *et al* 2014). Adaptive radiotherapy (ART) offers a promising avenue to adjust treatment plans over the course of therapy to account for the tumor’s dynamic nature (Yan *et al* 1997). Unlike standard RT, ART incorporates updated imaging data to optimize dose delivery during treatment, and has been successfully implemented against bladder, prostate, and head and neck cancer (Pos *et al* 2006, Ghilezan *et al* 2010, Schwartz and Dong 2011, McPartlin *et al* 2016, Castelli *et al* 2018, Morgan and Sher 2020, Hijab *et al* 2021). Moreover, it can be readily combined with dose delivery map-refinement strategies like dose painting by numbers (DPBN), where the dose is locally adapted based on measured or predicted information at the voxel level from imaging data (Bentzen 2005). This yields a personalized, non-uniform treatment plan delivered using techniques such as intensity-modulated radiation therapy (IMRT) which allows for steep radiation gradients across the targeted area.

DPBN relies on quantitative analysis of CT, MRI, or positron emission tomography (PET) data to provide quantitative insights into local biological heterogeneity, treatment resistance, hypoxia, or metabolism to identify regions that should be targeted for increased or decreased radiation dose. In (Pang *et al* 2023), Pang *et al* used cell density maps derived from diffusion-weighted (DW) MRI to locate areas of low tumor control probability in glioblastoma patients and perform local DPBN on those areas. They were theoretically able to locally increase the prescribed radiation dose delivered to the areas of high cellularity and raise the tumor control probability while maintaining constraints of radiation delivered to surrounding organs at risk. Moreover, the integration of DPBN-derived radiation plans into clinically available treatment planning systems has recently been established (Arnesen *et al* 2015, Orlandi *et al* 2016, Pang *et al* 2023), culminating in the phase III SPRECTRO GLIO trial which investigated the therapeutic benefits of boosting dose to specific areas guided by measurements of metabolic markers for glioblastoma multiforme (Laprie *et al* 2024). The study, which did not use ART, showed that despite dose-boosting plans up to 72 Gy being well tolerated by patients, overall survival did not increase for the overall cohort, prompting investigations into more sophisticated dose painting and treatment optimization pipelines. Thus, we posit that biology-based mathematical models can provide a framework for accurate and precise tumor forecasting to test the potential benefits of patient-specific treatment interventions, including simulating and evaluating the efficacy of different voxel-resolved dose plans (Yankeelov *et al* 2013, Rockne *et al* 2019).

Mathematical models of gliomas have been established that capture phenomena such as response to radiation treatment (Rockne *et al* 2009, Glazar *et al* 2020), angiogenesis (Hawkins-Daarud *et al* 2013, Hormuth *et al* 2019), mass effect (Garg and Miga 2008), and resection planning (Swanson *et al* 2008). For example, Corwin *et al* developed personalized, biologically-derived radiation plans by integrating a biomathematical model of glioma growth with a multi-objective evolutionary algorithm for IMRT optimization (2013). More specifically, spatiotemporally-resolved models relying on partial-differential equations (PDE) allow precise characterization of cell count and cell distribution at the voxel level (Jarrett *et al* 2020, Hormuth *et al* 2021, Lorenzo *et al* 2024). Those models are able to identify and predict areas of tumor progression or poor treatment response, and mark them as potential candidates for increased radiation dose. Of particular interest, Hormuth *et al* developed and evaluated a set of PDE models designed to represent different aspects of glioma behavior, including cell migration, proliferation, and response to treatment (Hormuth *et al* 2021, 2025). Patient-specific MRI data were used to initialize and calibrate the models to predict tumor growth with a median error in tumor volume under 2.5% with a two-species model describing the non-enhancing and enhancing areas of gliomas.

To build on these previous efforts, we employ dose painting and ART to refine, at the mid-point of RT, SOC dosing plans designed prior to treatment initiation to account for newly-observed changes and areas of progression. DW-MRI is used to estimate cell density maps to identify regions of growth and increased cellularity (Le Bihan *et al* 1986). Those areas are then subjected to a dose boost, yielding a new patient-specific and non-uniform dose delivery map for the remainder of RT. We then use the two-species glioma model proposed by Hormuth *et al* in (2021) to predict the effects of both the SOC and personalized dose maps. We hypothesize that the proposed optimized dose maps will lead to significantly greater predicted tumor control than the SOC uniform delivery maps one month after the completion of radiation treatment. Moreover, as a proof-of-concept, we investigate the potential physical deliverability of these plans and propose avenues for the clinical implementation of our adaptation procedure. This work contributes to the treatment of glioma by showing how adaptive RT combined with dose painting and biology-based mathematical models can better target tumor progression, offering a more precise and effective alternative to standard radiation plans.

## 2. Materials and methods

### 2.1. Patient cohort

The study involved 15 patients with histologically confirmed HGG as part of an institutional review board approved study at M.D. Anderson Cancer Center (NCT# 04771806). For this retrospective analysis, the institutional review board granted a waiver for informed consent. The proper regulations and guidelines were followed for all research protocols. MRI data were acquired at baseline and every week during the 6 weeks of radiation treatment. The clinical features of the patient cohort can be found in supplementary table S1. Patients were treated with the SOC recommendations following the Stupp protocol (Stupp *et al* 2005). They received a total dose of 60 Gy delivered in fractions of 2 Gy every weekday over 6 weeks. Concurrently, oral TMZ was administered at a dose of 75 mg kg^−1^ seven days a week. Following the end of RT, TMZ was orally administered for 5 d during a minimum of six 28 day cycles with a dose of 150–200 mg kg^−1^.

### 2.2. MRI data and processing

**Figure 1.**
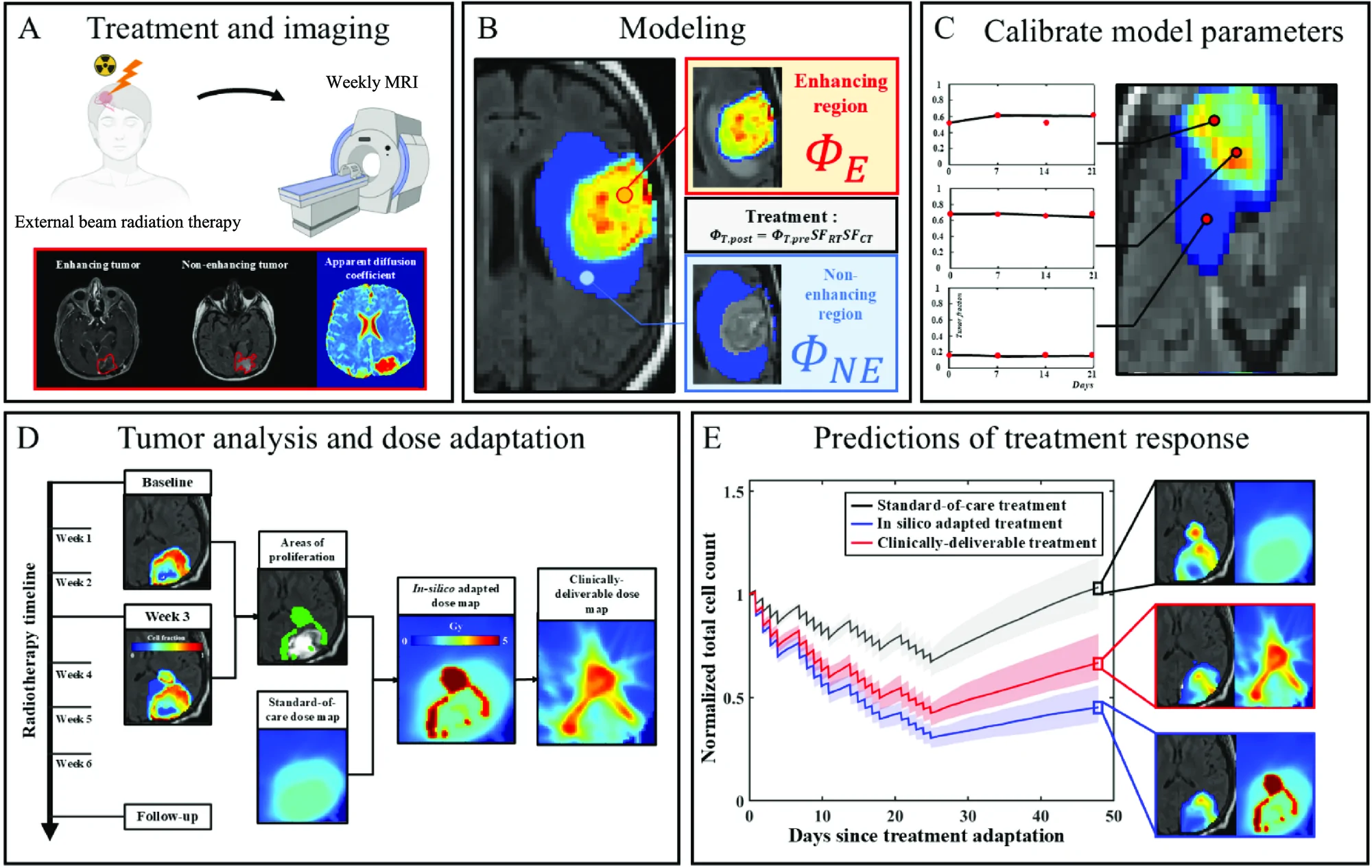
Flowchart of spatial adaptation of radiation to high-grade glioma patients. (A). High-grade glioma (HGG) patients are treated with standard-of-care radiation therapy and are imaged using quantitative MRI techniques. (B). A two-species model characterizing the enhancing and non-enhancing areas is used to capture the spatio-temporal development of HGG tumors. (C). The model is calibrated using cell density maps yielding parameter values for each voxel. (D). Areas of proliferation are identified by comparing cellularity at baseline and in the third week of treatment. A dose boost is prescribed to these areas to generate a patient-specific, personalized treatment plan. (E). Predictions of treatment response are performed for each dose plan using the two-species mathematical model to evaluate spatiotemporal tumor growth.

As a comprehensive description of the image acquisition process can be found in (Hormuth *et al* 2021) and (Miniere *et al* 2025), we provide only the main details of the acquisition and analysis protocol. Each MRI session collected: (1) a *T*_1_-weighted image, (2) a *T*_2_-FLAIR (fluid attenuated inversion recovery) image, and (3) DW-MRI data to construct apparent diffusion coefficient (ADC) maps (figure 1(A)). The baseline, mid-RT, and post-RT imaging sessions also included the injection of a (gadolinium-based) contrast agent. Pre- and post-contrast *T*_1_-weighted images were acquired on these visits, and the *T*_1_-weighted and *T*_2_-FLAIR images were used to segment the enhancing and non-enhancing regions of the tumor. Tumor segmentations were performed using a combination of semi-automated and manual approaches. The contrast-enhancing tumor regions were segmented manually on post-contrast *T_1_*-weighted images, while the non-enhancing (*T*_2_-FLAIR hyperintense) regions were initially identified using semi-automated intensity-based region growing and thresholding techniques, followed by manual refinement (*Raystation* built-in tools). All segmentations were performed by a trained expert and subsequently reviewed by a board-certified radiation oncologist to ensure accuracy and consistency. For imaging time points without contrast-agent administration, the enhancing and non-enhancing tumor regions correspond to those delineated at the most recent contrast-enhanced visit. As our mathematical model requires all images to be placed into the same imaging space, rigid registration was performed to align the images to the baseline *T*_2_-FLAIR scan using the *imwarp* and *imregtform* functions in MATLAB R2022a (Mathworks, Nattick, MA).

### 2.3. Mathematical model of tumor proliferation and invasion

Our model (Hormuth *et al* 2025) is based on a coupled set of reaction-diffusion equations that characterize tumor growth in the contrast-enhancing region (equation (1)) and the non-enhancing, *T_2_*-hyperintense region (equation (2)) (figures 1(B) and (C)):

\begin{equation*}\frac{{\partial {\Phi _{\mathrm{E}}}\left( {{\boldsymbol{x}},t} \right)}}{{\partial t}} = \nabla \cdot \left( {{D_{\mathrm{E}}}\left( {{\boldsymbol{x}},t} \right)\nabla {\Phi _{\mathrm{E}}}\left( {{\boldsymbol{x}},t} \right)} \right) + {k_{p,E}}\left( {\boldsymbol{x}} \right){\Phi _{\mathrm{E}}}\left( {{\boldsymbol{x}},t} \right)\left( {1 - \frac{{{\Phi _{\mathrm{E}}}\left( {{\boldsymbol{x}},t} \right) + {\beta _{NE}}{\Phi _{\mathrm{N}}}\left( {{\boldsymbol{x}},t} \right)}}{{{\theta _{\mathrm{E}}}}}} \right),\end{equation*}

\begin{equation*}\frac{{\partial {\Phi _{\mathrm{N}}}\left( {{\boldsymbol{x}},t} \right)}}{{\partial t}} = \nabla \cdot \left( {{D_{\mathrm{N}}}\left( {{\boldsymbol{x}},t} \right)\nabla {\Phi _{\mathrm{N}}}\left( {{\boldsymbol{x}},t} \right)} \right) + {k_{p,N}}\left( {\boldsymbol{x}} \right){\Phi _{\mathrm{N}}}\left( {{\boldsymbol{x}},t} \right)\left( {1 - \frac{{{\Phi _{\mathrm{N}}}\left( {{\boldsymbol{x}},t} \right) + {\beta _{EN}}{\Phi _{\mathrm{E}}}\left( {{\boldsymbol{x}},t} \right)}}{{{\theta _{\mathrm{N}}}}}} \right),\end{equation*}

where *Φ*_E_ and *Φ*_N_ respectively correspond to the volume fractions of the enhancing and non-enhancing tumor regions at 3D position ***x*** and time *t. β*_NE_ and *β*_EN_ are the inter-region competition terms, *D*_E_ and *D*_N_ are the diffusion coefficients, *k*_p,E_ and *k*_p,N_ are the proliferation rates, and *θ*_E_ and *θ*_N_ are the carrying capacities. Additionally, the diffusion coefficients vary spatially and temporally according to the local tissue mechanical properties as captured by the local von Mises stresses *σ*_vm_(***x****,t*):

\begin{equation*}{D_i}\left( {{\boldsymbol{x}},t} \right) = {D_{i,0}}\left( {\boldsymbol{x}} \right){\mathrm{exp}}\left( { - {\lambda _1}.{\sigma _{vm}}\left( {{\boldsymbol{x}},t} \right)} \right),\end{equation*}

where *D_i,0_* is the native tumor cell diffusion coefficient in the absence of stress, *λ*_1_ is the stress-tumor cell diffusion coupling constant, and *i* = *E* or *N*. The value of *σ*_vm_(***x****,t*) is computed at each iteration by solving the tissue displacement $\overrightarrow {\boldsymbol{u}} $ assuming linear elastic isotropic equilibrium as described by equation (4):

\begin{equation*}\nabla \cdot G\nabla \vec u + \nabla \cdot \frac{G}{{1 - 2v}}\left(\nabla \cdot \vec u\right)-{\lambda _2}\nabla \Phi {\mathrm{(}}{\boldsymbol{x}},t{\mathrm{)}} = 0.\end{equation*}

Literature values are used to assign tissue specific *G* and v for white matter and gray matter. Tumor cell response to chemoradiation is represented by a decrease in total cell count (i.e. the sum of ${\Phi _{\mathrm{E}}}\left( {{\boldsymbol{x}},t} \right)$ and ${\Phi _{\mathrm{N}}}\left( {{\boldsymbol{x}},t} \right))$ following treatment through equation (5):

\begin{equation*}{\Phi _{{\mathrm{T,post}}}}\left( {{\boldsymbol{x}},t} \right) = \Phi _{\text{T, pre}}\left( {{\boldsymbol{x}},t} \right)S{F_{RT}}\left( {{\boldsymbol{x}},t} \right),\end{equation*}

where ${\Phi _{\mathrm{T,pre}}}$ and ${\Phi _{\mathrm{T,post}}}$ are the pre- and post-treatment tumor cell fractions, respectively, and *SF*_RT_ and is the surviving fraction of tumor cells after a single dose of RT. We assume that *SF*_RT_ follows the linear quadratic model of response to RT proportional to tissue oxygenation:

\begin{equation*}{\mathrm{S}}{{\mathrm{F}}_{\mathrm{RT}}}\left( {{\boldsymbol{x}},t} \right) = {\mathrm{exp}}\left( { - \alpha .\frac{{{\mathrm{Dose}}\left( {{\boldsymbol{x}},t} \right)}}{{{\mathrm{OER}}\left( {{\mathbf{x}},t} \right)}}\left( {1 + \frac{{{\mathrm{Dose}}\left( {{\mathbf{x}},t} \right)}}{{\frac{\alpha }{\beta }.{\mathrm{OER}}\left( {{\boldsymbol{x}},t} \right)}}} \right)} \right),\end{equation*}

\begin{equation*}{\mathrm{OER}}\left( {{\boldsymbol{x}},t} \right) = \max \left( {{\mathrm{ER}}\left( {{\boldsymbol{x}},t} \right)} \right) - {\mathrm{ER}}\left( {{\boldsymbol{x}},t} \right) + 1,\end{equation*}

where *α* is the treatment sensitivity term, Dose(***x****,t*) is the RT dose (in Gy) delivered for a single fraction at voxel ***x*** and *α*/*ß* is the ratio of the linear and quadratic sensitivity terms fixed at a value of 10 Gy (Qi *et al* 2006). Finally, OER(***x****,t*) is the oxygen enhancement ratio, and ER(***x****,t*) is the enhancement ratio obtained by calculating the signal intensity ratio between the post- and pre-contrast *T*_1_-weighted images. As TMZ dosing remains the same throughout the six weeks of RT, we assume that the combined effect of RT and chemotherapy are captured though the calibrated *α* parameter (Barazzuol *et al* 2010). Numerical details regarding implementation and model calibration are described in (Hormuth *et al* 2021). A list of model parameters, as well as their definition, meaning, and how they are assigned can be found in supplementary table S2. We note that in a previous study, this model (i.e. equations (1)–(7)) was chosen as the most parsimonious (Akaike information criteria) from a group of 40 models integrating various representations of cell diffusion, growth, and treatment reaction (Hormuth *et al* 2021).

### 2.4. Dose map adaptation

We adapt the dose map at the mid-point of RT (MidRT). Areas of proliferation are identified by computing the proliferation map through the formula:

\begin{equation*}{k_{\mathrm{map}}}\left( {\overline {\boldsymbol{x}} } \right) = \frac{{{\Phi _{\mathrm{MidRT}}}\left( {\boldsymbol{x}} \right) - {\Phi _{\mathrm{baseline}}}\left( {\boldsymbol{x}} \right)}}{{{\Phi_{\mathrm{baseline}}}\left( {\boldsymbol{x}} \right)}},\end{equation*}

where *k*_map_ is the proliferation value at voxel position ${\boldsymbol{x}}$, ${\Phi _{\mathrm{MidRT}}}$ (${\boldsymbol{x}})$ and are the cancer cell fractions at position ***x*** at MidRT and baseline, respectively. Voxels are then classified as ‘proliferative’ if they display at least a 20% increase in cellularity from baseline to MidRT. Voxels containing no cells at baseline, but a positive number of cells at MidRT, are also classified as proliferative. The MATLAB function *imgaussfilt3* is then used to smooth and remove isolated voxels. The resulting map (figure 1(D)) identifies the voxels which will receive a dose boost. Dose painting is then applied by increasing the dose in those voxels by 3 Gy day^−1^, and smoothing (MATLAB function *imgaussfilt3*) is applied to avoid steep gradients in the dose map. The mathematical model is then evaluated forward from MidRT until the one-month post-RT follow-up time point to predict total tumor cellularity and cell distribution (figure 1(E)). As the proposed dose map was created *in silico*, it is important to note that this new dose map must be checked to ensure it can be physically delivered using clinically available linear accelerators.

### 2.5. Physical deliverability of *in silico* dose-adapted maps

As a proof-of-principle test, our adapted, patient-specific dose distributions were used to generate a radiation therapy plan that could be delivered using a typical linear accelerator. We imported the adapted dose distributions into the RayStation treatment planning system (version 12 A SP1, RaySearch Laboratories, Stockholm, Sweden). Treatment planning structures were constructed by segmenting the isodose levels in 10% increments from 0 to the maximum dose. We then computed cumulative dose volume histograms (DVHs) for each structure (brain stem, optic nerves, etc) using the optimized dose distribution. To build the deliverable version of the adapted dose maps, we added a series of minimum and maximum DVH optimization constraints to trace the shape of the original DVHs. Finally, we used the RayStation optimizer with three 6 MV coplanar intensity-modulated arc therapy beams and these optimization constraints to generate a deliverable dose distribution that maximally matched our adapted dose maps. Organs at risk were not used in the optimizer. Our goal was to accurately reproduce the dose distribution of the *in silico* dose-adapted plan. Further clinical development of this technique will need to include an optimization method that accurately reproduces the *in silico* dose-adapted plan as well as the physician’s clinical goals. For the remainder of this manuscript, the dose map generated *in silico* as described in section 2.4 above is referred to as the ‘*in silico* dose-adapted plan’, and the map obtained following adjustments for clinical deliverability as presented in this section is referred to as the ‘clinically deliverable plan’.

### 2.6. Statistical test and confidence intervals

After obtaining the predicted 3D tumor volumes at the one-month follow-up time point for the three dose maps and for each patient, a Mann–Whitney U test was performed to compare the resulting tumor voxel distributions from the SOC dose plan versus the *in silico* dose-adapted plan, and the SOC dose plan versus the clinically deliverable plan.

To generate confidence intervals on our predictions of cell counts, the parameters and their covariance matrix were used after calibrating the model to define a truncated multivariate normal distribution within the parameter limits. This distribution was sampled 100 times for each patient and the samples were used in forward evaluations of the model to generate confidence intervals for the tumor growth and treatment response predictions.

## 3. Results

**Figure 2.**
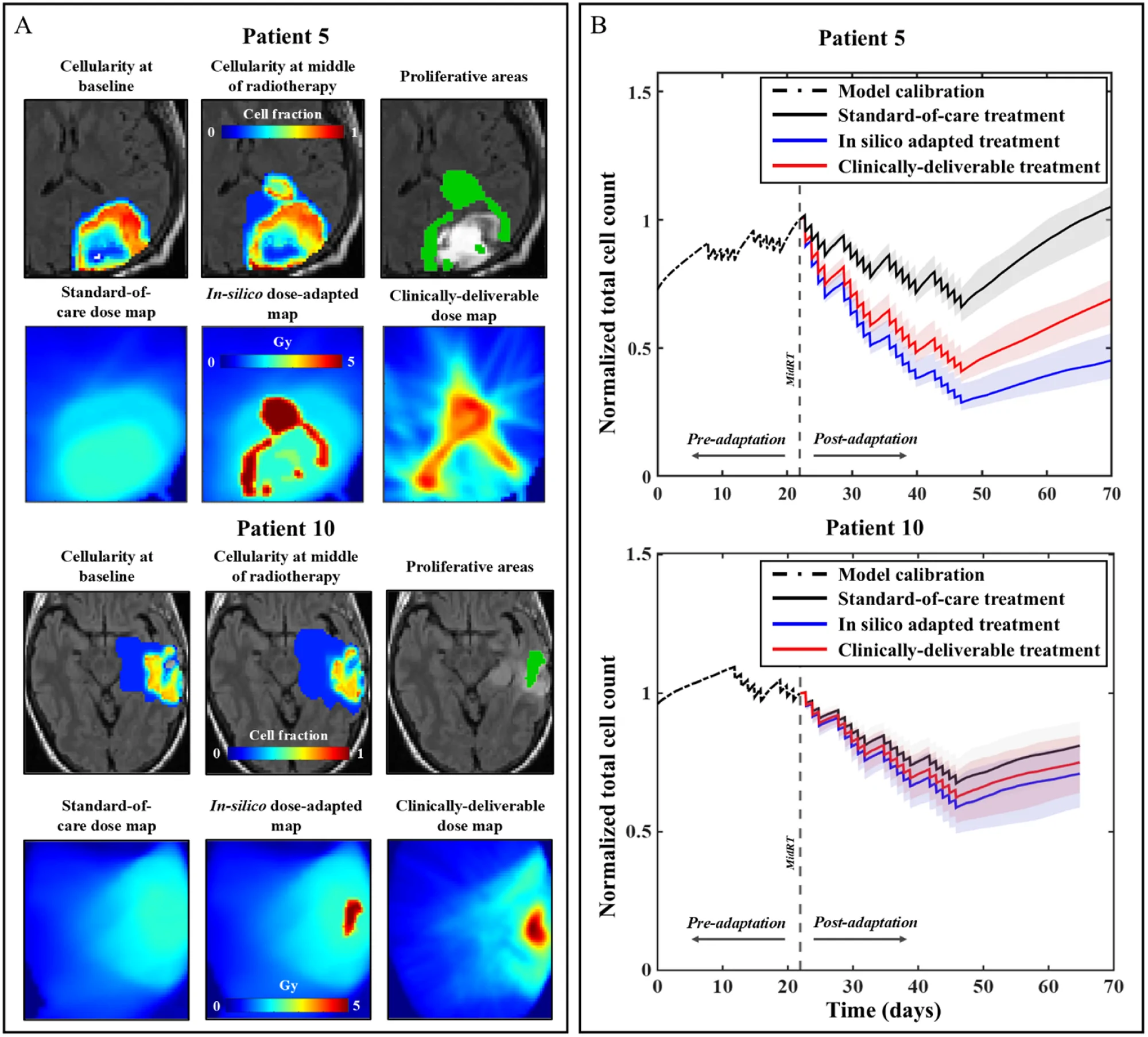
Treatment plan comparison for two high-grade glioma patients. (A). Cellularity at middle of treatment (MidRT) and baseline are used to identify areas of progression which are prescribed local dose boosts. Patient 5 shows greater tumor progression in the marginal area (i.e. the boundary zone of the tumor, where tumor cells interface with surrounding normal tissue), while growth is primarily observed in-field (i.e. the portion of the tumor located within the planned radiotherapy field) for patient 10. (B). Predicted tumor cell count trajectories from MidRT to the one-month follow-up visit, using the standard-of-care dose map (black line), *in silico* dose-adapted map (blue line), and clinically deliverable dose map (red line), with the model calibrated on data from baseline to MidRT. Shaded areas correspond to confidence intervals. Patient 5 shows greater predicted response to the adapted treatment than patient 10, principally due to the larger extent of the boosted area.

Figure 2 shows an example of our proposed ART pipeline applied to patients 5 and 10. Panel A illustrates the dose adaptation steps: comparison of tumor cellularity between baseline and MidRT, identification of proliferative areas, *in silico* dose-adapted map, and clinically deliverable map. For patient 5, the tumor shows substantial progression from baseline to MidRT as the total tumor cell count has increased by 24%. For patient 10, the observed area of proliferation is smaller, leading to a more restricted boosted region. Panel B presents model predictions of the tumor growth expected with each of three dose maps (i.e. the SOC map, *in silico* dose-adapted map, and clinically deliverable map), starting from the optimization time point (MidRT). On this panel, the mathematical model, calibrated on data acquired from baseline to MidRT, is then projected forward in time from MidRT until the 1 month follow-up visit. The black solid line corresponds to the prediction of treatment response with the SOC dose map. The blue line is the prediction obtained using the *in silico* dose-adapted map that does not account for if the dose can be clinically delivered. Compared with the predicted response at the one-month follow-up time point using the SOC dose map, the *in silico* dose-adapted map predicts a 55% reduction in tumor cell count for patient 5. Lastly, the red line represents the prediction of tumor growth using the clinically deliverable dose map. This dose map predicts a tumor cell count of 34% smaller compared to the forecast made with the SOC dose map for this patient. For patient 10, the smaller boosted region leads to predicted tumor cell count reductions of 12% and 7% with the *in silico* dose-adapted map and the clinically deliverable dose map, respectively. Further discussion on the causes of variabilities in predicted response between patients can be found in the following section.

**Figure 3.**
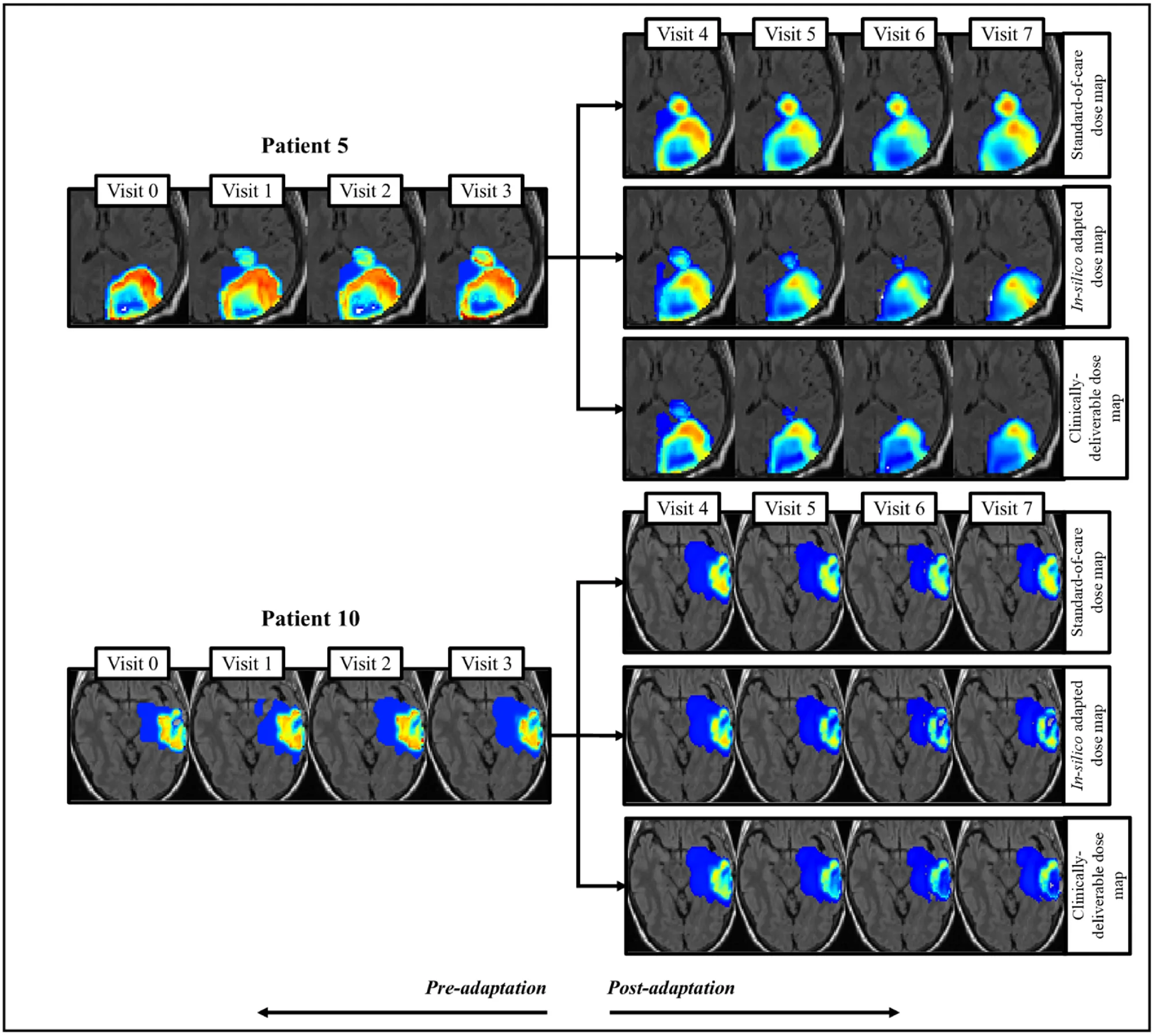
Predicted cancer cell distributions following dose adaptation for patients 5 and 10. Predicted cancer cell distributions following dose adaptation, illustrating the standard-of-care (SOC) dose map’s failure to control tumor progression (top row post-adaptation) compared to the enhanced tumor control of the *in silico* (middle row) and the clinically deliverable (bottom row) dose-adapted maps for patient 5. For patient 10, the *in silico* adapted and the clinically deliverable dose maps lead to reduced cellularity inside the gross tumor volume compared to the SOC map.

Figure 3 shows the cancer cell distribution corresponding to the predictions made with the three dose maps. For patient 5, note how the tumor progresses towards the center of the brain during the first 22 d of treatment (from visit 0 to visit 3), a phenomenon that the uniform and static SOC dose map fails to properly treat (top row). The model prediction shows that the *in silico* dose-adapted map, by giving a dose boost to this specific area of progression, successfully prevents the invasion (middle row), as does the clinically deliverable dose map (bottom row). In the case of patient 10, the *in silico* dose adapted and the clinically deliverable dose maps both yield slightly decreased tumor burdens in the tumor area compared to the SOC map.

**Figure 4.**
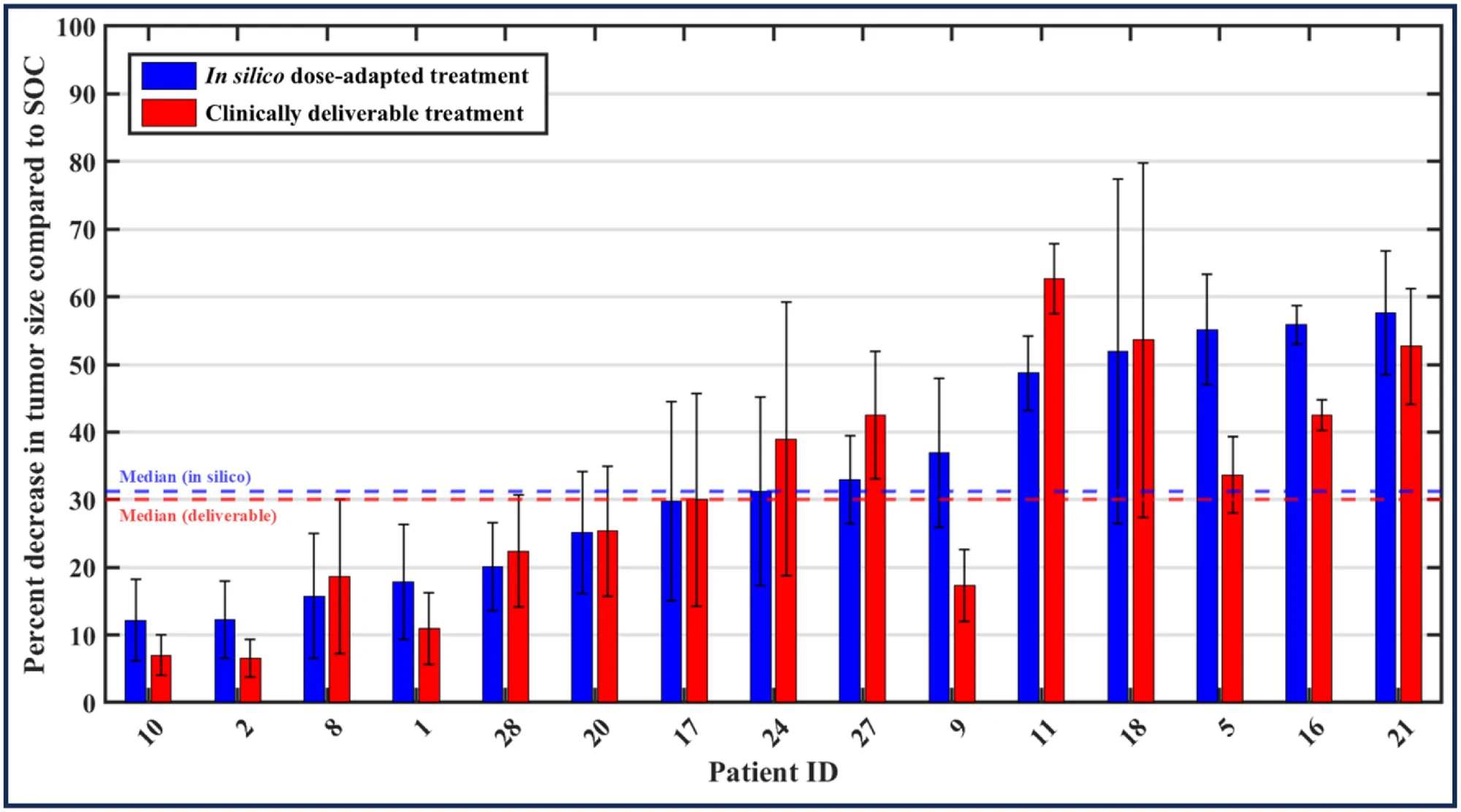
Comparison of predicted tumor size decrease between *in silico* dose-adapted plans and clinically deliverable plans across the patient cohort. Box plot showing the percentage decrease in tumor burden at one-month post-radiotherapy for the *in silico* dose-adapted plans (blue) and clinically deliverable plans (red) relative to the standard-of-care dose map, highlighting a median decrease of 31% and 30%, respectively. Large differences in expected tumor burden reduction are observed across the cohort, with patients exhibiting poor response to the prescribed dose boosts unlikely to benefit from the proposed adaptation protocol. All decreases in tumor burden are statistically significant. Error bars represent standard deviations.

Figure 4 shows the predicted tumor burden reduction obtained for the *in silico* dose-adapted map (blue) and the clinically deliverable dose map (red) compared to the SOC prediction for the entire patient cohort. One month following the conclusion of RT, the median decrease was 31% using the *in silico* dose-adapted map, and 30% with the clinically deliverable maps. We note the strong inter-patient heterogeneity in predicted tumor burden reduction, ranging from 12%/7% (*in silico* dose-adapted map/clinically deliverable map) for patient 10, to 57%/53% for patient 21. The differences between the tumor voxel distributions obtained between the adapted plans, and the SOC plans at the one-month follow-up time point are statistically significant (p < 0.05) for all patients.

Moreover, for the *in silico* dose-adapted plans, the cohort exhibited a median reduction of 31% (interquartile range: 31%). For the clinically deliverable plans, the median reduction was 30% (interquartile range: 24%). For both groups, a Wilcoxon signed-rank test against zero yields a *p* value of 6.1 × 10^−5^ confirming that both plans are statistically different from 0%. Bootstrap resampling (i.e. repeatedly sampling the patient cohort with replacement to generate a distribution of median tumor reductions) at the patient level (10 000 iterations), yielding 95% confidence intervals of [25.5% and 41.9%] for the *in silico* dose-adapted plans and [22.5% and 39.6%] for the clinically deliverable plans. Importantly, these intervals do not include zero, confirming that the observed tumor reduction represents a consistent cohort-wide improvement over the SOC rather than being driven by a subset of patients. Note that to further isolate the impact of our targeted dose painting approach, we implemented an additional dose plan which corresponds to a homogeneous boost across the entire dose map, designed so that this new map and the *in silico* dose-adapted map have the same total dose. Supplementary figure S3 presents the results for a representative case (patient 5, the same patient presented in figure 2). Over the cohort, the homogeneous dose boost led to a predicted median decrease of 11% (interquartile range: 14%) compared to the 31% obtained with the *in silico* dose-adapted map.

## 4. Discussion

We have developed a clinical-computational pipeline to personalize the delivery of radiation to account for observed changes in tumor cellularity for a cohort of 15 HGG patients using widely available multiparametric MRI data. By identifying areas of tumor progression by week 3 of treatment, we increased the dose given to those areas by 3 Gy per daily fraction, which yielded statistically better predicted control of the regions of increased cellular activity for all patients. To ensure patient safety and feasibility of delivery, our theoretical radiation plans were then refined by a trained medical physicist using a clinically available treatment planning software. To establish the clinical benefit of our adapted plans, a two-species reaction-diffusion model (Hormuth *et al* 2021, Miniere *et al* 2025) was calibrated individually on each patient using estimates of cellularity obtained via DW MRI. The enhancing and non-enhancing regions of the tumor were delineated using *T*_1_-weighted and *T*_2_-FLAIR images, thereby integrating tumor heterogeneity into our modeling efforts, with local variations in proliferation captured by our spatially-resolved calibrated proliferation map. Using this model, predictions of treatment response can be performed for the SOC, non-adapted dose map, and our personalized, adapted maps. Our forecasts show that our adapted plans are hypothesized to outperform the SOC plans with statistically significant decreases in tumor burden for all patients at one-month post-therapy. We also investigated a homogeneous boost across the dose plan to reach the same total dose as our proposed spatially-targeted plans. At the cohort level, this yielded a statistically lower reduction in total tumor burden than our proposed plans (*p*= 0.00350, showing that selectively boosting areas of high proliferation yields a definitive advantage over uniformly increasing the dose across the target volume.

Our contribution aligns and expands on recent efforts to personalize radiation treatment using imaging-guided computational tools. For example, Lipkova *et al* utilized a Bayesian framework integrated with MRI and 18 F-fluoroethyl-tyrosine positron emission tomography to estimate cell growth and generate patient-specific clinical target volumes and associated dose boosting plans (2019). Similarly, Zhang *et al* (2025) proposed an approach to estimate mathematical model parameters from a single MRI measurement using a physics-informed neural network. Their model predicts tumor cell density and validates glioblastoma infiltration against tumor recurrence data, thereby aiding the tailoring of RT plans to the individual. As their approach relies on a single imaging timepoint prior to treatment, we extend this concept by integrating time-resolved measurements allowing us to explicitly target and account for tumor progression.

It is important to note that there are a range of benefits experienced from patient to patient across the cohort; that is, some patients are hypothesized to experience a reduction in tumor burden by as much as 57% (e.g. patient 21 with the *in silico*, dose-adapted plan), while others may realize only modest reductions of 12% (e.g. patient 10). Those latter cases can exhibit a very low sensitivity to radiation as captured by a high calibrated SF_RT_ (i.e. surviving fraction to RT) value. Due to this low radiosensitivity, we predict that it is unlikely that such cases would benefit from a targeted dose boost. Moreover, lower response to an optimized dose plan compared to the SOC can also be explained by minimal tumor progression at the mid-point of RT. If only minor tumor growth is observed by MidRT, then the corresponding adapted dose plan would only provide a dose boost to a very restricted area, leading to minimal differences in predicted total tumor cell count obtained using the SOC and the adapted plans.

Two particular areas require further investigation to establish the practical utility of the proposed pipeline. First, we applied a substantial boost of 3 Gy day^−1^ in addition to the initially-prescribed 2 Gy day^−1^, resulting in daily fractions of 5 Gy day^−1^ to the target areas in the post-dose adaptation phase of the treatment. We acknowledge that this daily dose exceeds the typical recommendations of glioma, and we emphasize that the primary aim of this work is to present a systematic method to identify candidate areas for dose boosting through mid-treatment imaging, rather than optimizing the boost size itself. As an exploratory analysis, we performed a sensitivity analysis considering additional boost levels of 1 Gy day^−1^ and 2 Gy day^−1^, alongside the 3 Gy day^−1^ boost presented in the main manuscript. This analysis reveals a clear dose–response relationship, with increasing boost magnitude leading to progressively greater reductions in tumor burden for both the *in silico* and clinically deliverable plans (supplementary figure S4). The 3 Gy day^−1^ boost leads to the largest mean reduction, and the clearest separation from the 0% baseline. In contrast, lower boost levels, particularly 1 Gy day^−1^, show reduced efficiency and overlap with 0% in the case of the clinically deliverable plans, reflecting poor or limited predicted benefit. We note that the stronger separation with 0% at the highest dose levels is expected as larger doses directly increase tumor cell death in our modeling framework. Recall that the goal of this work not is not to advocate for a specific boost size, but rather to help demonstrate the utility of mathematical modeling in estimating the benefits of various treatment approaches. While the 3 Gy day^−1^ boost provides the clearest predicted benefit, such dose escalation must be carefully balanced against normal tissue toxicity and organ-at-risk constraints to maximize the therapeutic ratio.

**Figure 5.**
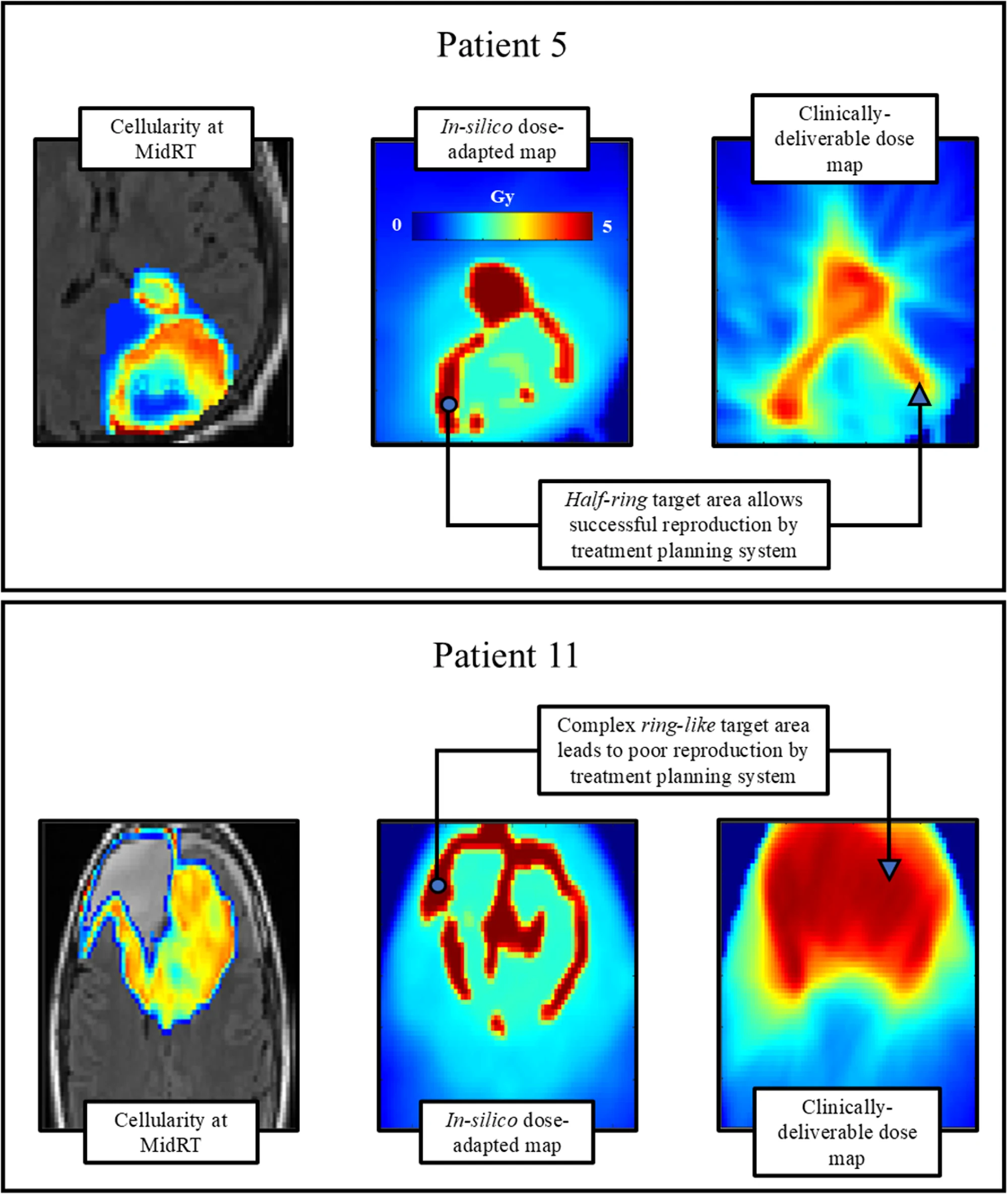
Examples of adjustments of *in silico* adapted plans using a clinically available treatment planning system. For half-ring or non-ring target geometries as prescribed for patient 5, current beam planning algorithms allow successful reproduction of the *in silico* dose-adapted plan for delivery by linear accelerators. This approach fails in the case of more complex, ring-like target structures (as seen for patient 11), which leads to over-boosting over the entire tumor area.

The other area that requires further investigation is related to guaranteeing that the proposed adapted plans are clinically viable. That is, the adapted plans must be delivered using currently-available beam path generation schemes without increased risks to healthy tissues and structures due to the applied dose boost. Key challenges remain regarding the precision of beam modulation to distribute radiation in the designated areas of progression while limiting off-target effects. In general, this can be successfully achieved for half-ring- or non-ring-shaped targets as presented in the clinically deliverable plan controlled by the medical physicist in figures 2 and 4 for patient 5. However, complex full-ring or *ring-like* distributions may exceed the capabilities of widespread conventional RT planning algorithms and beam optimization routines (patient 11 on figure 5).

Current linear accelerators rely on multileaf collimators to shape radiation distributions, with a limited resolution that might not adequately reproduce the finer spatial variations prescribed in our adaptive maps. Limitations regarding multileaf collimator precision are known to lead to discrepancies between desired and delivered dose maps, especially in areas with steep dose gradients (Losasso 2008). Further refinement of beam planning techniques tailored to these maps are thus warranted to mitigate the risk of overdosing nearby tissues. However, there are emerging technologies that promise superior accuracy than existing systems. For example, stereotactic surgery uses multiple sources to focus radiation with sub-millimeter precision and can target small, intricate structures (Housepian 1971, Kano *et al* 2015). Proton therapy can also be considered to deposit radiation following the Bragg peak, allowing precise energy distribution at the tumor depth while sparing healthy tissues beyond the target’s boundaries (Weber *et al* 2020). Thus, the dose maps presented in this contribution should be regarded as a proof-of-concept demonstration of the computational-to-clinical translational pipeline, as our deliverability assessment relied only on whether the proposed dose plans could realistically be implemented on a conventional linac. To make the approach more clinically realistic, we would need to include organ-at-risk dose constraints, target coverage, conformity index, and homogeneity index, and quantitative criteria for failure cases. Such investigations are ongoing. Our analysis nonetheless allows potential conceptual categorization of patients based on expected benefit from spatially-targeted adaptive RT. Notably, using the cohort median reduction as reference (∼30% reduction) we find: high-predicted benefit patients (IDs: 17, 18, 16, 21) typically exhibit larger predicted tumor reduction (median for those patients: 55%), consistent with more active proliferative regions identified at mid-treatment and/or higher radiosensitivity (median surviving fraction to RT and number of proliferative voxels: 0.92 and 7445, respectively). Conversely, low-predicted benefit patients (IDs: 10, 2, 8, 9, 28, 20) had more limited predicted improvement (median for those patients: 17.5%), with smaller regions of proliferation and reduced radiosensitivity (median surviving fraction to RT and number of proliferative voxels: 0.96 and 1344, respectively). Lastly, delivery-challenging cases (IDs: 11, 24, 27) correspond, as stated earlier, to situations where the *in silico* dose-adapted plan contains ring-like or complex boosted geometries, difficult to reproduce using conventional linear accelerators. We note that this classification does not imply that dose optimization should not be attempted on these patients, but rather points to current technical limitations and potentially suggests that future advances in treatment planning and delivery systems could enable more accurate reproduction of the proposed boosted dose distributions for difficult tumor geometries. Future research should thus focus on developing innovative optimization routines that align the proposed dose distributions with the technical constraints of existing linear accelerators, or on leveraging the performances of next-generation systems. Furthermore, timing of dose boosting and a more refined fractionation schedule can also be integrated into a mathematical platform such as the one proposed, allowing to investigate alternative treatment planning strategies on a patient-by-patient basis (Muayad *et al* 2021).

## 5. Conclusion

We have presented a computational-clinical platform to systematically adapt and personalize RT dose plans for HGG. This work bridges a critical gap in glioma treatment by showcasing the potential of ART and biology-based mathematical modeling to account for and address the dynamic nature of tumor progression during RT. Given the notoriously poor prognosis for HGG, our findings suggest that personalized dosing strategies could lead to better tumor control notably in areas of high cellularity, and thus potentially extend patient survival beyond what is obtained through current SOC practices.

## Data Availability

The data cannot be made publicly available upon publication because they contain sensitive personal information. The data that support the findings of this study are available upon reasonable request from the authors. Supplementary data available at: https://doi.org/10.1088/1361-6560/ae9687/data1.
